# Software Defined Radio-Based Wireless Sensing System

**DOI:** 10.3390/s22176455

**Published:** 2022-08-26

**Authors:** Yihan Xu, Reza K. Amineh, Ziqian Dong, Fang Li, Kayla Kirton, Michael Kohler

**Affiliations:** 1 Department of Electrical and Computer Engineering, New York Institute of Technology, New York, NY 10023, USA; 2 Department of Mechanical Engineering, New York Institute of Technology, Old Westbury, NY 10023, USA

**Keywords:** software defined radio, soil sensing, surface acoustic wave sensor, wireless sensing

## Abstract

In this paper, we investigate the application of using software-defined radio (SDR) and surface acoustic wave (SAW) device for wireless measurement of the response of in situ sensors. SDR uses software to realize different communication functions. After collecting the magnitude and phase of the response at discrete frequencies, we apply inverse Fourier transform to analyze the time domain responses which, in turn, allows for monitoring the changes of the response of the in situ sensor. We employ microwave signal flow graph concepts to improve the quality of the received signals. Comparing the normalized results obtained by SDR with those obtained from a commercial vector network analyzer (VNA), we demonstrate that the results are sufficiently close, and the SDR-based experiments can provide satisfactory measurement of the in-situ sensors. The objective is to eventually employ this wireless measurement system for soil nutrient sensing.

## 1. Introduction

Wireless sensor technology is currently one of the most rapidly growing research fields. It has a great potential in the following applications: Internet of Things (IoT) [1], military [2], agriculture [3], medicine [4], transportation [5], and security [6]. With the development of wireless sensor networks based on IoTs [7], low-energy wireless sensors have become a significant research trend. However, designing a small, low-complexity, high-precision wireless sensing system is yet to be developed in many applications. Therefore, in this paper, we address this pressing need by developing a robust, compact, accurate, and low-cost wireless sensing system that can be used for reading sensors wirelessly. The main objective is to apply this system for soil sensing applications [8,9]. In agriculture, soil nutrient levels are important factors for precision farming. Healthy soils have the ability to improve water and air quality, while contaminated soils not only lead to reduced soil quality and farmland productivity, but also endanger food, ecological security, and human health [10,11]. Therefore, soil information collection and dynamic monitoring have become the direction of future soil research.

To obtain accurate reading from in situ sensors (sensors embedded inside the soil) wirelessly, systems have been proposed in [12,13,14,15] which employ commercial Ground Penetrating Radar (GPR) and surface acoustic wave (SAW) reflective delay line components. A typical SAW reflective delay line consists of an Interdigital Transducer (IDT) and multiple reflectors lined up on the surface of a piezoelectric substrate. The device does not contain a battery and can be interrogated wirelessly and passively by an interrogation unit, such as radar. During the operation, the antenna connected to the IDT receives electromagnetic pulses transmitted from the interrogation unit. Due to the inverse piezoelectric effect of the substrate, the IDT converts the electric signal into a SAW. Then, the SAW propagates towards reflectors distributed in a characteristic pattern and is reflected at each reflector. Finally, the IDT converts the returning waves into electrical signals, and the antenna sends them back to the interrogation unit. If an external impedance sensor is connected to a reflector (i.e., sensing reflector), the acoustic reflection properties of this sensing reflector are changed, altering the reflected signals’ amplitude, frequency, and delay time. Thus, the impedance value of the external sensor can be measured with such wireless and passive sensor systems.

Although commercial GPR has been employed to current wireless SAW sensor system [12,13,14,15], commercial GPRs are expensive, cumbersome, and inflexible to adapt to different environments. In such systems, the parameter settings such as spectrum and sampling rate are often fixed and cannot be programmed to adjust to different needs. In addition, some commercial GPRs have limited sensing capability due to time-based drifts [16].

In order to scale up the wireless reading of in situ sensors such as those embedded inside the soil, a low-cost and portable device is required to replace commercial GPR. To meet this requirement, Software Defined Radio (SDR) can be considered a feasible solution due to its low cost, low power profile, and small size. Compared with the traditional radio structure, most of the functions of SDR are realized by software, thus reducing the hardware complexity. SDRs can be reprogrammed for different purposes, making them more flexible than traditional radars.

In this study, we propose a novel portable wireless sensing system that uses SDR along with a SAW device to acquire the response of in situ sensors. We aim at developing this system for detection of nutrients in soil in the future. While the SAW device can measure different parameters such as temperature, moisture, etc. [17], here, it is mainly used to separate the reflections from the environment (interferences) and the reflections from the SAW device in the received radar signal. In this work, for controlled experiments and evaluations, we do not use an external polymer sensor and instead we connect variable resistive loads to the SAW device. The sensor can be polymer-based such as the one proposed in our previous work [18] which is PEDOT for nitrate detection [19]. In our previous work [20], we have observed that the resistance of PEDOT sensor changes from 0 Ohm to 150 Ohms under various nitrate concentrations. Thus, we use a similar resistance range for the variable resistor board connected to the SAW device replacing the PEDOT sensor. Furthermore, in this work, the SAW device and the variable resistor board are measured in the air (not inside the soil) due to the low power of the utilized SDR device.

We use SDR to send and receive signals and obtain spectral information from the SAW device. The resistance changes of the polymer due to the variance in the soil nutrient concentration is detected by the magnitude and phase changes in the reflected signals, as shown in our previous study [20]. We convert the frequency domain data to the time domain to analyze the received signals and measure the change in the resistance of the polymer sensor. In the proposed system, we use LimeSDR-mini as the SDR system [21]. Compared to other SDR-based wireless sensing projects that use higher frequencies (e.g., 915 MHz) [22,23,24], LimeSDR-mini allows for operating at lower frequencies such as 250 MHz [25]. This allows for deeper penetration of the RF signals in the soil for characterization and sensing. These things considered, our system has a narrow bandwidth, low structural complexity, and high precision. Lastly, we employ microwave signal flow graph concepts to improve the quality of the received signals.

The remainder of the paper is organized as follows. Section 2 presents the proposed framework of our wireless sensing system. Section 3 introduces the experimental setup of the proposed system. Section 4 presents our results and Section 5 concludes the paper.

## 2. Proposed Wireless Sensing System Using SDR and SAW Devices

**Figure 1:**
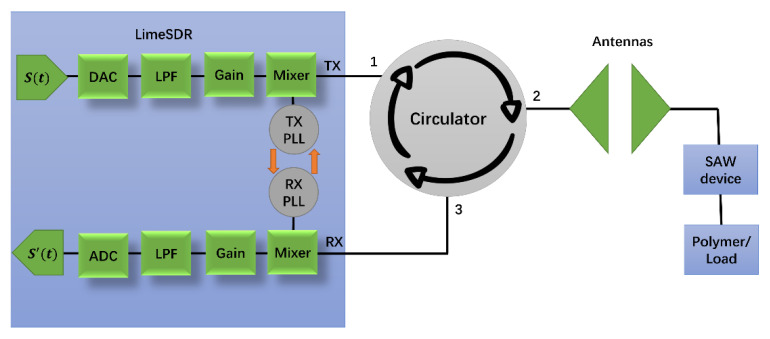
Proposed wireless sensing system including the LimeSDR-mini, circulator, two antennas, SAW device, and polymer (variable resistive load). On the circulator, the numbers 1, 2, and 3 mean port 1, port 2, and port 3, respectively. The circulator only allows microwave path from port 1 to port 2, port 2 to port 3, and port 3 to port 1.

The proposed system is composed of a transmitter and a receiver implemented on a LimeSDR-mini, a circulator connected to a transmitting antenna, a receiving antenna connected to a SAW device, and a polymer sensor (here, we employ a variable resistor board to emulate the response of the polymer sensor). The block diagram of the wireless sensing system is shown in Figure 1. The LimeSDR-mini system includes an analog-to-digital signal converter (ADC), digital-to-analog converter (DAC), low-pass filter (LPF), and mixer blocks to send signals in a selected frequency range. The mixer provides the output signal frequency from two input signal frequencies. Moreover, LimeSDR-mini has the functionality of automatically setting the phase-locked loop (PLL) to ensure the output signal’s phase is related to the phase of the input signal.

The system uses the time division duplex (TDD) mode [26] so that Transmit (TX) and Receive (RX) modules can share the same antenna to reduce the complexity of the structure and to improve the spectral efficiency. The circulator allows the signal to flow in one directional from the transmitter to the transmitter antenna and prevents the reflected signal from entering the transmitter port in the other direction (enabling the transmitter and receiver to use the same antenna). The TX of LimeSDR-mini is connected to port 1 of the circulator, and port 2 is connected to the transmitter antenna. The antenna transmits the signal from the LimeSDR-mini and receives the reflected signal from the SAW device. When the resistance of the polymer changes, the received signal changes accordingly. The received signal is then passed to the RX through port 3 of the circulator.

The wireless sensor system in Figure 1 uses LimeSDR-mini as the signal generation source. It adopts an IQ (in-phase and quadrature) modulation structure, and over time, it generates the time series signal *S*[*t*] [27]:

(1) $$S\left[t\right]=I_{R}cos\left(2\pi f_{c}t\right)+jQ_{R}sin\left(2\pi f_{c}t\right)\;$$

where $f_{c}$ is the operation frequency and $I_{R}$ and $Q_{R}$ denote the in-phase carrier and quadrature-phase carrier, respectively.

LimeSDR-mini provides IQ measurements of the signal. To measure the time delay, we need to convert the measurements from frequency domain to time domain using the measured magnitude and phase data. According to the digital IQ modulation, we can get magnitude and phase under the frequency at the time through (1).

**Figure 2:**
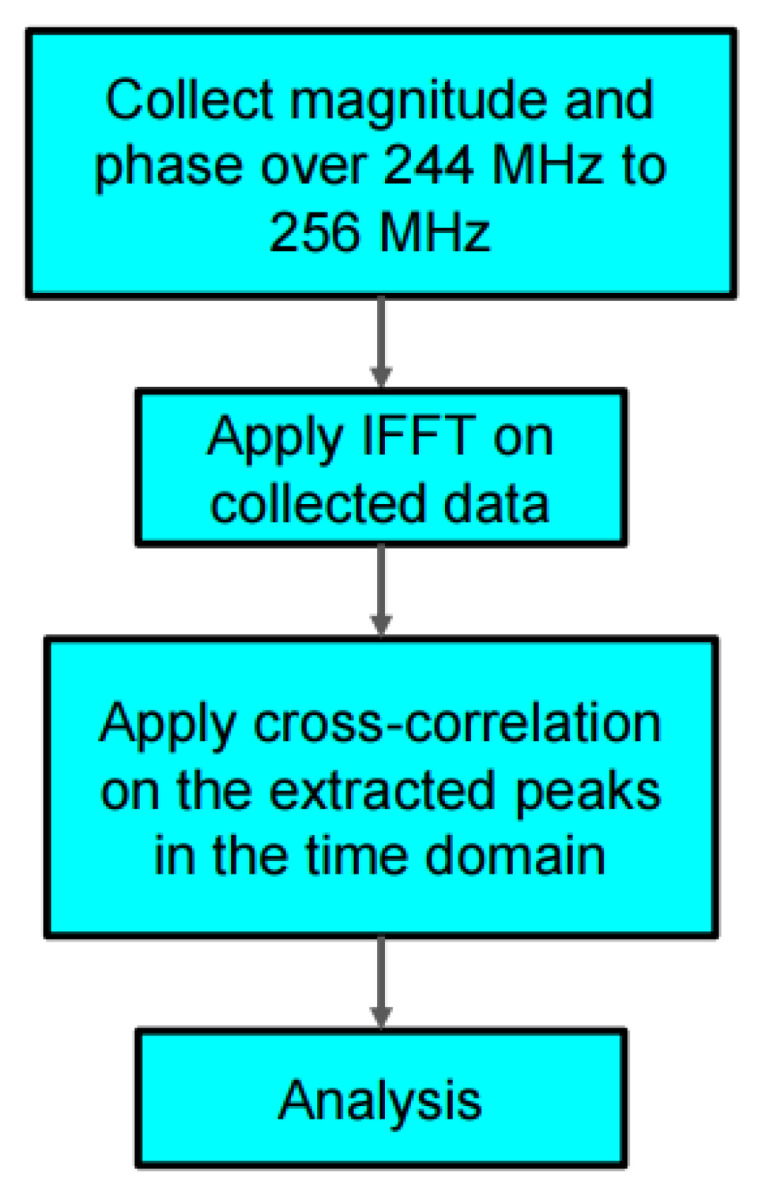
Flow chart of the proposed measurement system.

Figure 2 shows the flow chart of the data collection and analysis process for our proposed system. We collect the magnitude and phase data in the frequency domain and at 1921 samples over 244 MHz to 256 MHz. Then we apply inverse Fourier transform to convert the frequency domain data to time domain for calculating the time delay caused by the SAW device and resistive load (replacing the polymer sensor).

Since LimeSDR-mini does not have a tight channel-to-channel alignment between TX module and RX module, the result of each phase measurement is different. Here, we propose a phase measurement method to resolve the channel alignment issue. In (1), we set the values of *I* and *Q* to be fixed (for example, *I* = 0 and *Q* = 1) and thus the phase of TX does not change with time. Therefore, the phase change between TX and RX is also fixed.

Like in VNA measurements, calibration is needed to compensate the effect of systematic errors, cables, and connectors. Although SDR ports may face self-interference, low output power and calibration can reduce the effect of leakage between the ports. Short circuit calibration is performed by connecting a standard short component to port 2 of the circulator to measure the magnitude and phase, denoted by $R_{Short}$ and $\varphi_{Short}$, respectively. We denote the measured magnitude and phase of the device under test (DUT) as $R_{DUT}$ and $\varphi_{DUT}$, respectively.

Then, the calibrated magnitude and phase results, denoted as ∆M and ∆φ, respectively, are obtained as:

(2) $$∆M=R_{DUT}\left(\mathrm{dB}\right)-R_{Short}\left(\mathrm{dB}\right)\;$$

(3) $$∆\varphi =\varphi_{DUT}-\varphi_{Short}$$

**Figure 3:**
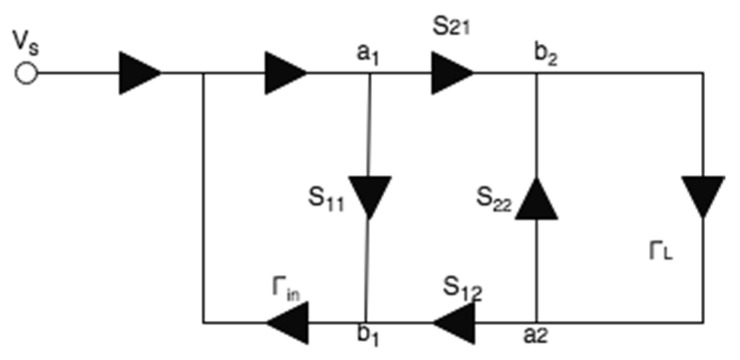
Two-port network used to simulate wireless communication with SDR and SAW device. *S*-parameters belongs to antennas, $\Gamma_{in}$ is the reflection coefficient measured on the LimeSDR-mini side, $\Gamma_{L}$ is the reflection coefficient from the SAW device [28].

At 250 MHz, the power of LimeSDR-mini is about −7 dBm which is much lower than that of a commercial GRP (for which the power is about 18 W). Consequently, under the wireless transmission condition, because of the free-space path loss and antennas’ reflection losses, the strength of the reflected signals may be insufficient for analysis if using this utilized SDR for measuring sensors embedded inside the soil. Thus, the use of additional amplifiers can be considered for soil sensing applications. Moreover, to improve the signal quality, we propose a method to remove the effect of the free-space loss, the reflection loss, and the near-field coupling of the antennas. Figure 3 shows a two-port network representing the two antennas network, for which, port 2 is connected to a load with reflection coefficient of $\Gamma_{L}$ is related to the input impedance seen toward the input port of the SAW device. $\Gamma_{in}$ is the reflection coefficient measured on the LimeSDR-mini side. In this figure, $S_{ij}$ (*i* and *j* = 1, 2) denote the scattering parameters of the two antennas. The relation between $\Gamma_{in}$, $\Gamma_{L}$ and the antenna’s *S*-parameters is as follows [28]:

(4) $$\Gamma_{L}=S_{11}+\frac{S_{12}S_{21}\Gamma_{L}}{1-S_{22}\Gamma_{L}}$$

Using the measured antennas’ *S*-parameters, $\Gamma_{in}$, and (7), $\Gamma_{L}$ can be obtained as:

(5) $$\Gamma_{L}=\frac{\Gamma_{in}-S_{11}}{\Gamma_{in}S_{11}-S_{11}S_{22}+S_{12}S_{21}}$$

**Figure 4:**
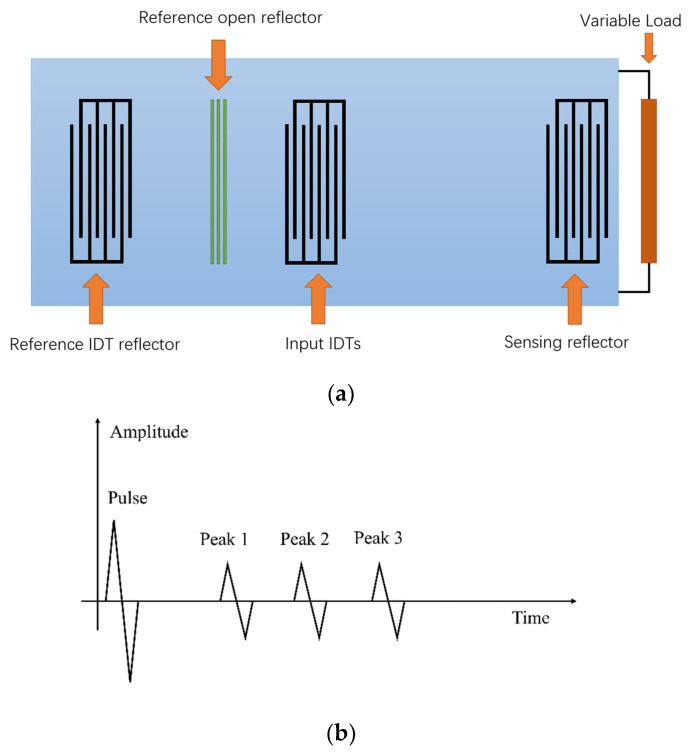
SAW device’s design and its expected result in time domain: (**a**) the utilized SAW device for which IDTs and reflectors have 22 fingers; electrode material is aluminum and substrate material are 128° YX $\mathrm{LiNbO}3_{3}$ and (**b**) the expected received signal in the time domain. Peak 1 is the reflection from the reference open reflector, Peak 2 is a reflection from the reference IDT reflector, and Peak 3 is the reflection from the sensing reflector.

Figure 4a shows the design of the SAW device used in our experiments. The SAW device consists of an input IDT and three reflectors. The reflectors connected to the external impedance sensors are sensing reflectors, while the ones without external sensors are used as references to compensate for the environmental effects. The two reference reflectors are on the left side of the input IDT, and the sensing reflector is on the right side of the input IDT. The three reflectors will generate three reflection peaks in the time domain response. Figure 4b illustrates the time domain variations of the transmitted and reflected pulses. Since the propagation speed of surface acoustic waves is almost 100,000 times slower than that of the electromagnetic waves, the reflected signals from the SAW reflectors can be well separated from the reflections received from the surrounding environment (interferences travel with the speed of electromagnetic waves while desired responses travel with the speed of acoustic waves). Peak 1 is the reflection from the reference open reflector. Peak 2 is the reflection from the reference IDT reflector. Peak 3 is the reflection from the sensing reflector. Without considering the environmental effects, such as temperature, the two reference reflectors are fixed, and the time delay between Peak 1 and Peak 2 is also fixed. When the resistance of the polymer changes, the time delay between Peak 3 and Peak 1 or Peak 2 will change [29,30]. To assess the time delay between the peaks, we employ cross-correlation. For example, for computing the cross-correlation between Peak 1 and Peak 3, we take the magnitude in the time domain and pick three points which are the beginning, maximum, and ending for each peak region. For this purpose, we set an empirical threshold level much lower than the maximum peak values such that for each peak we get two points on the right and left side of the peak. The threshold can be considered as the static standard on time domain, based on the threshold, we can calculate the correlation between each peak on the time domain. The two points for Peak 1 are $I_{11}$ and $I_{12}$ and for peak 3 are $I_{31}$ and $I_{32}$. Then, in order to calculate the cross-correlation between them, we first apply interpolation to have the number of samples between $I_{11}$ and $I_{12}$ and between $I_{31}$ and $I_{32}$. After getting the cross-correlation between Peak 1 and Peak 3, we normalize it and find its maximum lag and repeat the same steps for Peak 1 and Peak 2 to use it as a reference lag. The lag between Peak 1 and Peak 3 minus the reference lag is the amount of the time delay.

## 3. Experimental Setup

Our experimental setup includes a LimeSDR-mini [21] with a frequency range from 10 MHz to 3.5 GHz and the maximum bandwidth of up to 40 MHz. The two channels correspond to TX and RX. It has a dimension of 69 mm × 31.4 mm in size making it portable. It is programmable through GNURadio and C++ API.

**Figure 5:**
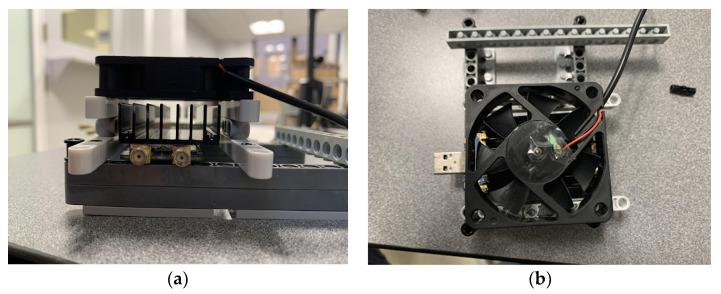
LimeSDR-Mini cooling system: (**a**) side view showing the heatsink and the fan and (**b**) top view showing the fan.

**Figure 6:**
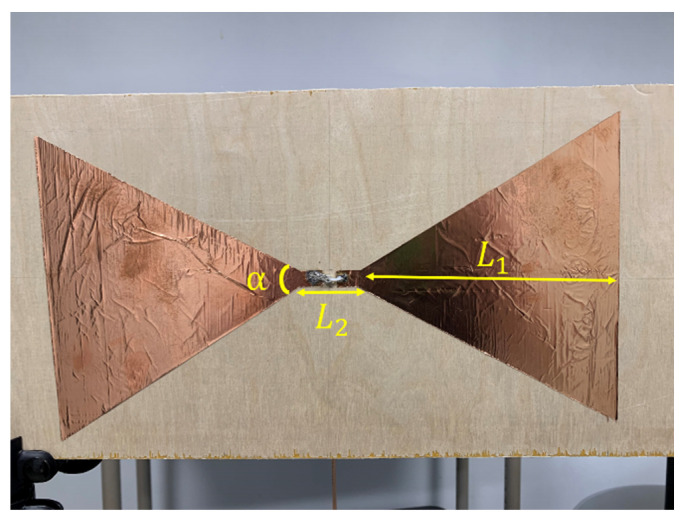
Bow-tie antenna designed around center frequency of 250 MHz, α = 60 degrees, $L_{1}$ = 185 mm, and $L_{2}$ = 20 mm.

**Figure 7:**
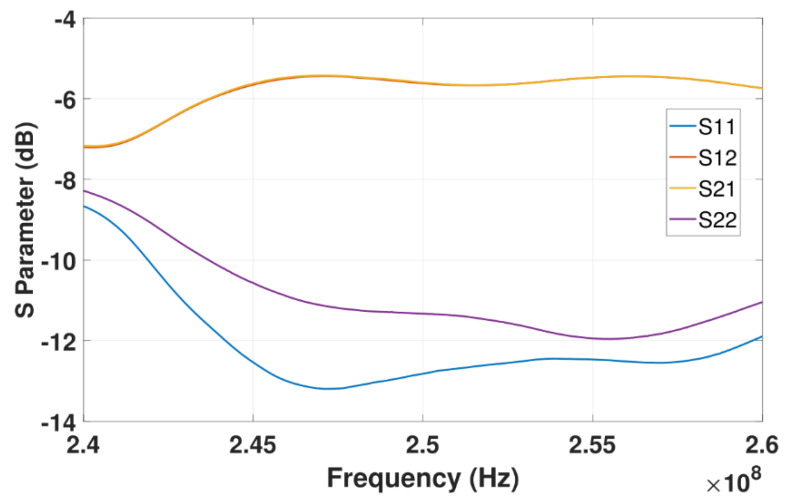
Bow−tie antennas’ *S*−parameters.

Our system is designed to operate at 250 MHz center frequency to allow sufficient penetration of the RF signals into the soil (up to 1 m of depth when used in practice). To validate our results, first, we compare the measurements between the LimeSDR-mini to those measured by a VNA. In the experiments, when the running time increases, the temperature increase of the SDR produces additional noise. In the frequency domain, as the frequency increases, the measured magnitude response has a large deviation from the one measured by VNA. Therefore, in practice, the LimeSDR-mini chip is coated with CPU thermal grease and a heat-sink is attached to it. We also place fans above and below the SDR to accelerate the heat dissipation as shown in Figure 5. As for the circulator in Figure 1, we employ RFLC101M20M30 Circulator from RF-Lambda which covers the 250 MHz operating frequency. Regrading antennas, we design bow-tie antennas to operate at a center frequency of 250 MHz. The antenna radiator is copper, its base is wood, and it is fed by SMA connector. Figure 6 shows the structure of the antennas. The antenna has a symmetrical design; *α* denotes the angle of the flare which is 60 degrees; *L*_1_ denotes the length of each triangular element which is 185 mm; *L*_2_ denotes the gap between two triangular elements which is 20 mm. Figure 7 shows the antenna’s *S*-parameters when they are placed face-to-face and the distance between them is 10 cm. Although in soil sensing applications, the distance between the two antennas may be larger than 10 cm in some cases (when the sensor is placed deeper inside the soil), adding an additional amplifier to the system could allow for increasing such distance. Furthermore, to reduce the near-field coupling effect for the antennas, we use the method proposed in the previous section that allows for reducing the effect of antennas including their coupling on the measured reflection coefficient. In the frequency range that we tested from 244 MHz to 256 MHz, *S*_11_ and *S*_22_ of the antennas are lower than −10 dB which indicates satisfactory radiation properties. Figure 7 shows the transmission *S*-parameters (*S*_21_ and *S*_12_), indicating that there is almost 5.5 dB transmission loss from one antenna to the other. In practice, we use LimeSDR-mini to measure antennas *S*-parameters under a fixed distance for wireless sensing.

**Figure 8:**
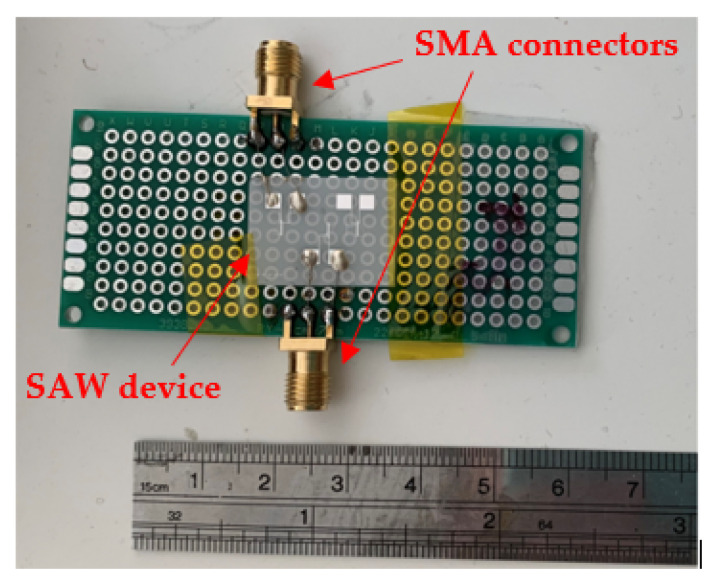
SAW device and its size. It uses SMA type to connect antenna and variable load board [20].

The frequency range of the designed SAW device in Figure 8 is from 240 MHz to 260 MHz. There are two reference reflectors on the piezoelectric material to be employed as the reference time delay in the time domain analysis of the measured responses. Table 1 shows the design parameters of the SAW device [20].

**Figure 9:**
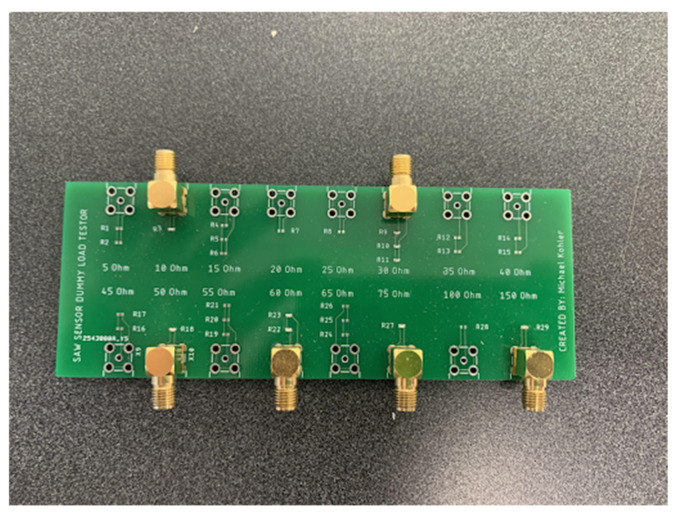
Variable load board.

In order to simulate the resistance variation for the polymer sensor connected to the sensing port of the SAW device, we designed a board including surface mount device (SMD) resistors as shown in Figure 9. The values of the resistors are within the range of 5 Ω to 150 Ω representing the range of polymer sensor’s resistance change [17].

**Figure 10:**
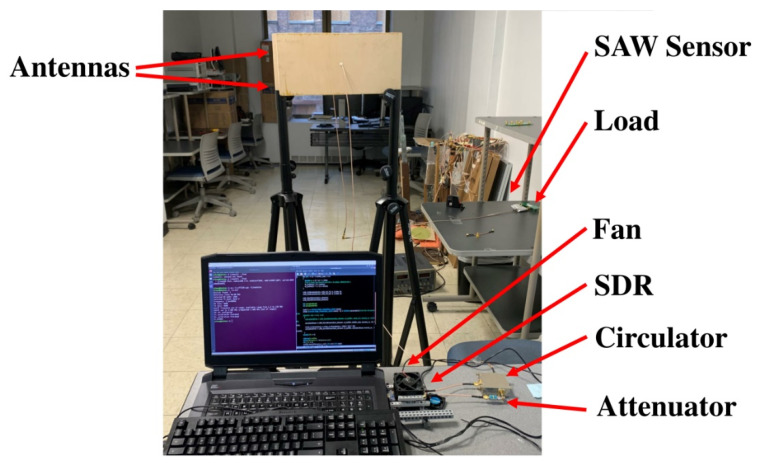
Experimental setup.

Figure 10 shows the experimental setup for the proposed system. Compared to the diagram in Figure 1, a 6 dB attenuator is added between the TX and port 1 of the circulator. The use of an attenuator in the TX path helps to provide a fixed impedance and control output power, which will reduce the effects of load changes and improve the measurement accuracy. We did further investigations about the effect of attenuator and observed that when using that, the output signal of the TX has much weaker harmonics at the attenuator output. Furthermore, without an attenuator, the quality of the received signal degrades and for a given transmitted signal, we cannot get constant magnitude and phase at the receiver. In our tests, a 6 dB attenuator was giving satisfactory results. A laptop computer is used to operate the SDR and store the data.

## 4. Experimental Results

**Figure 11:**
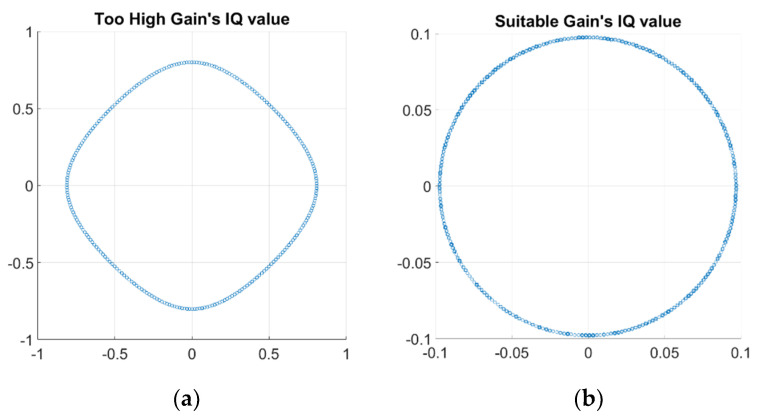
The received IQ plots for: (**a**) high gain setting and (**b**) suitable gain setting.

In the parameter design of LimeSDR-mini, the gain of TX and RX can be set within the range of [0, 72] dB. However, in the experiments, the range of gain that can be set is much smaller than the provided range as shown in Figure 11. The reason that the received signals with high gain setting and low gain setting provide two different curves is due to the LimeSDR-mini receiver’s sensitivity. In [31], the SDR system has been used for medical imaging and they report similar gain setting problem. According to our observation which is consistent with those in [31], both TX and RX gain settings need to be adjusted in order to avoid signal saturating and clipping problems. In our experiment, we performed a large number of tests to find the suitable gain range under our experimental conditions. The suitable range of TX gain is 0 to 61 dB and the suitable range of RX gain is 0 to 23 dB.

The experiment is run in the Linux environment, and we use C++ to compile and implement all the functions. Table 2 lists the parameter design of LimeSDR-mini. The sampling rate is set at two values: 1 MHz and 10 MHz. 1 MHz is used to measure the magnitude and 10 MHz is used to measure the phase. A low sampling rate can be used to measure a stable magnitude value. For the phase, the sampling rate must be higher than the RC time constant for the low pass filters. That is why the sampling rate is 10 MHz for the phase measurement. In order to obtain a precise time delay, the frequency step should be small. In addition, since the LimeSDR-mini is powered by the laptop USB, the signal is weak. Even if the TX gain is set to 60 dB, the signal strength is relatively low. So, here, the distance between the two antennas is as small as 10 cm to ensure that the SAW device can receive a signal of sufficient strength.

**Figure 12:**
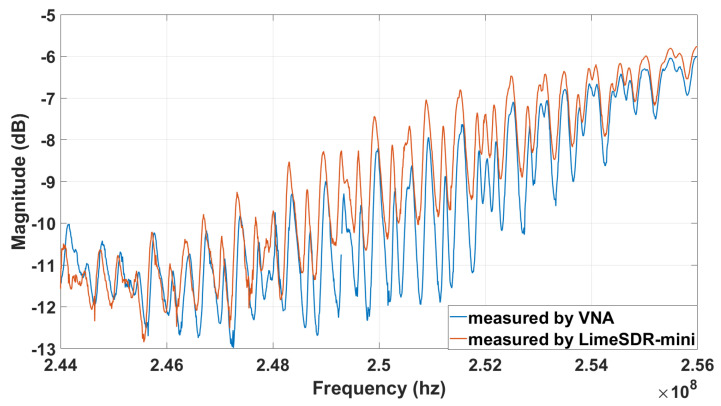
Magnitude response in the frequency domain measured by VNA and LimeSDR−mini from 244 MHz to 256 MHz.

In the experiment, we use VNA measurements as reference. We use KeySight E506SA ENA for measuring responses over the frequency range of 244 MHz to 256 MHz with number of samples of 1921 and the averaging of 16. The reason for such a large number of samples is that the SAW device has sharp and oscillatory responses in the frequency domain. Thus, to capture such response accurately, we need to collect a large number of frequency samples. Here, our transmit signal’s bandwidth is 10 MHz (LimeSDR-mini’s bandwidth setting in Table 2). However, the frequency bandwidth that we are going to measure is 12 MHz which is from 244 to 256 MHz. Figure 12 shows the comparison of the magnitude response in the frequency domain for the SAW sensor obtained by LimeSDR-mini and VNA measurements. When the frequency increases, compared with the VNA’s magnitude response, LimeSDR-mini’s magnitude response is shifted upwards. The reason for this shift is the thermal noise from the device. The cooling system can alleviate but does not eliminate the thermal noise.

**Figure 13:**
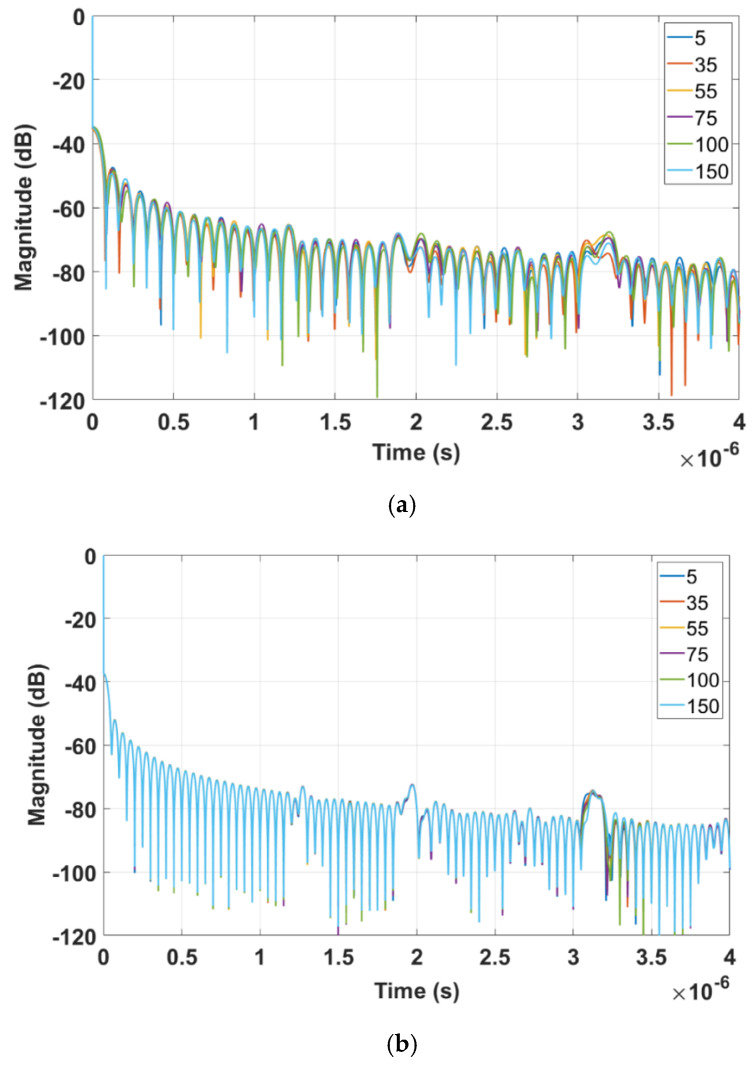
Magnitude of the time domain response after applying inverse Fourier transform to the frequency domain data: (**a**) measured by LimeSDR−mini and (**b**) measured by VNA.

**Figure 14:**
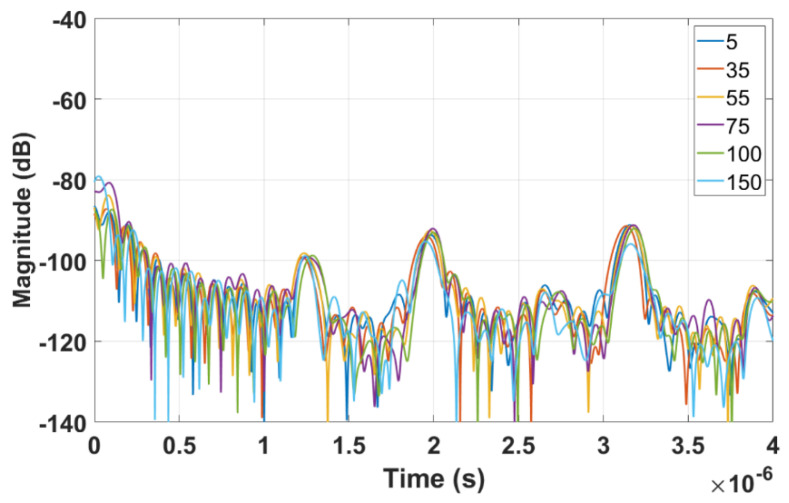
Time domain response from LimeSDR−mini data after compensation of the free space propagation loss, antennas’ reflection loss, and antennas’ near−field coupling.

**Figure 15:**
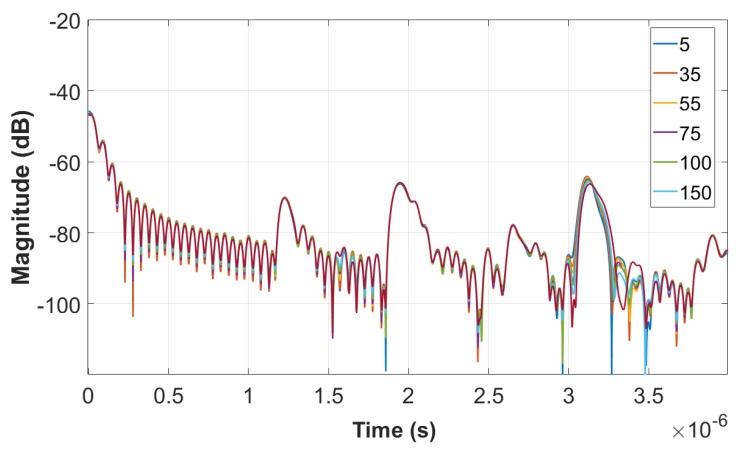
Time domain response from the measured VNA data after compensation of the free space propagation loss, antennas’ reflection loss, and antennas’ near−field coupling.

Figure 13 shows the time domain plots obtained by LimeSDR-mini and VNA for different resistances of the load (unit: Ohm), respectively. The three peaks in the time domain of LimeSDR-mini are too weak to analyze. Referring to the results of VNA, although three obvious peaks can be seen, the signal strength is not large enough to perform a reliable analysis of the time delays. Thus, we employ the measured *S*-parameters of the antennas and the technique proposed in Section 2 to reduce the influence of the antennas on the responses. Figure 14 and Figure 15 are the time domain responses of the LimeSDR-mini and VNA after compensation of the signal due to free path propagation loss, antennas’ reflection loss, and antennas’ near-field coupling as proposed in Section 2. For these restored responses, the intensities of the three peaks are enhanced to a level that can be analyzed more reliably. In particular, the levels and the width of the peaks have been restored such that they can be robustly detected and analyzed for the time delay calculation as described in Section 2.

To track the change in the time domain responses when varying the resistance values (corresponding to the polymer sensor), the cross-correlation methodology that was described in Section 2 is employed to find the time delay of peak 3 relative to peak 1 and peak 2.

**Figure 16:**
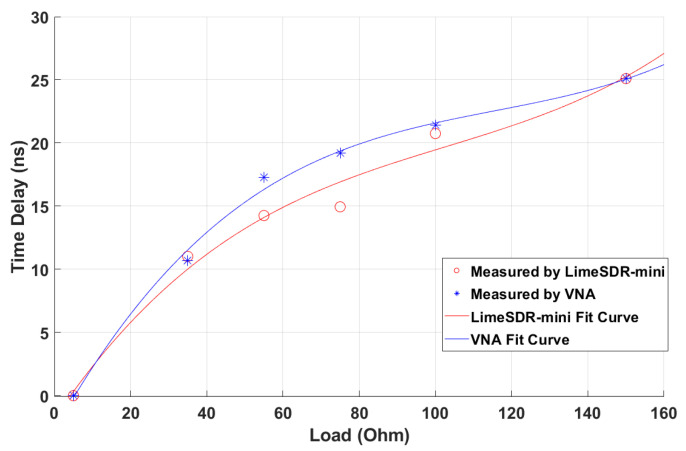
The normalized time delay results for LimeSDR-mini with the absolute time delay scaled based on the VNA and their 3rd fit curve.

Figure 16 shows the measured time delay plots compared with the results of cubic curve fitting for both VNA and LimeSDR-mini. It is observed that the value of the time delay increases with the increase of the resistive load. The rate of increase is sharper for load values below 60 ohms. Furthermore, Figure 16 shows that the load values can be unambiguously obtained from the measured time delays. This, in turn, provides a robust a reliable means to wirelessly measure the polymer sensors embedded inside the soil or any other in situ sensor for which the response is in the form of the resistance change.

Further, we quantify the sensitivity of this system defined as:

(6) $$sensitivity=\frac{∆T}{∆R}$$

where ∆T is the change in the time delay and ∆R is the change in the resistance of the load. For both LimeSDR-mini and VNA, the *sensitivity*’s range is within [0.05, 0.367] and the largest values of sensitivities correspond to loads below 40 ohms. Moreover, when the load increases, the *sensitivity* decreases. To sum up, the *sensitivity* of the SDR is similar to that of the VNA at the head and tail of the load range, but in the middle of the load range, SDR’s *sensitivity* is lower than VNA’s.

In order to evaluate the goodness-of-fit, we introduce Root Mean Square Error (RMSE) and R-square. RMSE is used to illustrate the degree of dispersion of the samples. When doing the nonlinear fitting, the smaller RMSE is, the higher the similarity. R-square represent the fitted regression effect. The closer R-square is to 1, the better the fitting result is. In Figure 16, we use a 3rd degree polynomial fit curve since it has provided satisfactory results in [20]. For the VNA and LimeSDR results, the R-squares are 0.9957 and 0.9821, respectively, and the RMSEs are 0.03726 and 0.07295, respectively. It can be seen from the fitted curves that when the load increases, the time delay increases. When comparing the result of LimeSDR-mini and VNA, the R-square is 0.8573 and the RMSE is 0.0074. It shows that LimeSDR-mini and VNA time delay results are close verifying the measurement accuracy by the SDR system.

## 5. Conclusions

The wireless sensing system constructed by SDR and SAW device in this paper offers a low-cost (total cost under $200), portable, high-quality in-situ sensor measurement system with the main application aimed at soil sensing. We presented the following issues for such system: complete calibration, parameter settings, hardware structure, and signal enhancement based on the microwave signal flow graph concepts. To validate the performance of the proposed system, we compared the obtained results from the SDR system with those from VNA. The normalized time delay curves obtained from both SDR and VNA are close. The proposed system can accurately distinguish the time delay corresponding to different resistive loads. To improve the signal-to-noise ratio for the wireless measurement of sensors at larger distances, RF amplifiers can be employed. Furthermore, the portability of SDR makes it possible to place the system on a drone for soil sensing over a large field (when many passive SAW devices and polymer sensors are scattered over a vast field).

In the future, we aim at using this system for implementing field test using the polymer sensors for soil nutrient sensing. For this purpose, a comprehensive signal loss analysis needs to be performed when the SAW device and the polymer sensor are buried inside the soil. Furthermore, the effect of other environmental factors such as temperature and moisture need to be considered. Furthermore, the sweeping time for collecting the SAW device’s response and antenna’s *S*-parameters is up to 6 h. This measurement time is not prohibitive for soil sensing applications (the property of the soil does not change rapidly, and fast monitoring is not required). However, to reduce the measurement time for other applications that require faster reading of the sensors, faster SDR systems can be employed along with narrower bandwidth, and a lower number of frequency samples.

We have demonstrated that combinations of the low-cost SDR, antennas and SAW device can provide satisfactory results compared with VNA. However, there are still some shortcomings. LimeSDR-mini’s dynamic range is about 60 dB which is worse than dynamic range of VNAs which is typically around −100 dB. In addition, the measurement time is much slower than VNA as described above. Lastly, it works based on a 12 ADC resolution. While for our tests, it can provide satisfactory results, for low frequency test (like the 250 MHz frequency we use) it has a frequency shift that needs to be compensated manually.

Finally, in our experiments, the environment temperature is a very important factor that influences the time delay results. It has been observed that as the temperature increases, for the same load, the time delay increases. Thus, in practice, the environment temperature needs to be monitored and compensated employing prior knowledge about its effect.

## Figures and Tables

**Table 1:** SAW device parameters.

Parameters	Specification
Wavelength (λ)	15.6 μm
Electrode material	Al
Metallization ratio	0.5
Electrode thickness	434 nm
Aperture	100 λ
IDT and reflector finger number	22
Distance from reference open reflector to reference IDT reflector	100 λ
Distance from input IDTs to reference open reflector	150 λ
Distance from input IDTs to sensing reflector	400λ

**Table 2:** LimeSDR−mini parameters setting.

Title 1	Title 3
Frequency Rang	244 to 256 MHz
Sampling Rate	1 MHz/10 MHz
Step Frequency	6250 HZ
Number of Frequency Points	1921
Bandwidth	10 MHz
TX Gain	60 dB
RX Gain	20 dB
Digital Low Pass Filter	100 Hz

## Data Availability

Not applicable.
